# The Negro, Major Shufeldt’s Wonderful Book*“The Negro, a Menace to American Civilization,” by R. W. Shufeldt, M. D., Major Surgeon Medical Department U. S. A. (retired); R. G. Badger, Pub., Boston

**Published:** 1908-04

**Authors:** 


*"The Negro, a Menace to American Civilization," by R. W. Shufeldt, M. D., Major Surgeon Medical Department U. S. A. (retired); R. G. Badger, Pub., Boston.

*effect* of a *cause* that should never have existed. We are always dealing with *effects*,—in treating disease, in taking care of insane and paupers, in punishing and caring for criminals,—instead of preventing them. "Our hindsight is better than our foresight," as I heard one express it more forcibly than elegantly.

It was a great mistake our forefathers made in ever importing negroes. The descendants of the first twenty black cannibals brought to America, and the thousands added by the infamous slave trade, encouraged by the United States government, now number about ten millions, and, according to Drs. Shufeldt, Graves, Thomas and others, are leavening the loaf, degrading the race, by admixture with the American people until it threatens to undermine and destroy American civilization. It is too late to put up the gap,—the mischief is done; we are confronted by a menace to our race integrity, and what are we going to do about it? Great truths are sometimes expressed and carry conviction when told in simple language, or by homely simile. You fill your bath tub with clean water and foolishly let in a small stream of muddy water. Soon your bath will be ruined unless you can dip out or strain out the mud,—or throw it all out. Mr. Strauss is now adopting the filtering process to remedy the fearful mistake of overwhelming immigration of the undesirable. It seems to me that the only rational course with reference to the negro is to segregate them,—put every one on a reservation like we did the Indians (that is Graves' idea), and keep them there, or, better, deport the entire race. Let them go back to their kin, and stay there. The latter is recommended by Dr. Shufeldt, as the alternative to race deterioration and ultimate destruction, or, meantime, a race war. Dr. Shufeldt's book is simply terrific. He tells the truth in the most forcible and direct manner; it is science, there is no sentiment in it; and shows that we are confronted by a real danger of immeasurable magnitude,—a danger to our national or, rather, race integrity. He says that of the ten million blacks in America fully one-half have more or less white blood in their veins, and that no power on earth can prevent the white man from fruitful intercourse with the females. He says the time will come when no person will be sure that a taint of black blood is not in his veins. The book reads like a romance, but is cold facts scientifically discussed. I wish these truths could be brought home to the sentimentalists who weep over the poor, down-trodden African, and they be made to realize the danger to their descendants.

But Congress will do nothing. The patriots are all dead. Altruism is unknown to our politicians. They are for the most part like Madame Pompadour: "After *me,* the deluge." It is a real pleasure to read this wonderful book. Dr. Shufeldt treats the negro question from the standpoint of biology and ethnology,—gives the general principles of interbreeding and shows how the admixture of inferior blood leads to decadence, decay and extinction; treats of hybridization, atavism, heredity, etc. He then discusses the passion and criminality of the negro, rape, lynch law and other questions, and, finally, sees no remedy for the evil except in entire separation of the races. The book is profusely illustrated by original photographs or drawings by the author himself. The price of the book is $1.50.

---

Bureau of Public Health and Marine Hospital Service of the United States; Annual Report of the Surgeon General for the Fiscal Year 1907; Government Printing Office, Washington, D. C. Thirty-ninth Annual Report of the "Service."

This is an interesting and important publication. It is especially interesting at the present time because of an awakened public sentiment that "our greatest national asset" (the public health, Roosevelt) is not sufficiently safeguarded; that it is, in fact, hampered by inadequate appropriation and inadequate departmental service,—and that necessarily, being administered by a bureau, in one of the departments, there are many details of sanitary science that can not be applied to the prevention of disease; they are too numerous. And this sentiment is finding expression through the medical and the lay press by writers and speakers in collaboration with the Association for the Advancement of Science, an immense body of the most distinguished and learned men in America, in every branch or department of learning, and their special committee of one hundred on Federal legislation. It is the opinion of this committee that the power and scope of action of the Public Health Service should be increased; that, instead of there being one bureau with several functions, there should be separate bureaus under one head, covering the entire field of sanitation or "preventive medicine," and that there should be no stint in the appropriation. The Surgeon General has done wonders with his cramped organization and small powers and means, and it emphasizes the necessity for more scope and power. For instance, immigration should come under the Public Health supervision. At least, there should be a Bureau of Immigration, one of Quarantine and Inspection, one of Animal Industry, one of Education, one of General Sanitation, one of Foods, Drugs and Liquors, one of Infant Hygiene, one of Architecture and Engineering, and, indeed, many other and every other subject that has a bearing on the health of the nation. It can readily be seen how this arrangement would protect against polluted water, adulterated liquor and foodstuffs; would reform building in the matter of ventilation, plumbing, lighting, heating and drainage, and diminish the danger of fires, etc. In fact, sanitary science should be applied to the prevention and removal of the thousand and one sources of disease and injury. The report makes a good showing—"considering." The law of July, 1902, enjoining supervision of vaccine, sera and toxins has been enforced with excellent results, and something has been done in milk and dairy inspection, locally. Space will not permit a more detailed notice of the report. The subject of patent medicines—a prolific factor in the production of ill health, insanity and drunkenness—should constitute a bureau in itself, and should, along with the very general practice of adulterating liquor to cheapen it receive a large share of the attention of the Public Health officials. Abortifacients should be suppressed, and it should be a penal offense to advertise them. See any "religious" paper, and many not so "religious."

